# Acupuncture for Chronic Pain-Related Insomnia: A Systematic Review and Meta-Analysis

**DOI:** 10.1155/2019/5381028

**Published:** 2019-06-24

**Authors:** Fushui Liu, Jianyu You, Qi Li, Ting Fang, Mei Chen, Nana Tang, Xiaojun Yan

**Affiliations:** ^1^College of Acupuncture-Moxibustion and Tuina, Jiangxi University of Traditional Chinese Medicine, Nanchang, China; ^2^Jiangxi Normal University, Nanchang, China; ^3^The Affiliated Hospital of Jiangxi University of Traditional Chinese Medicine, Nanchang, China; ^4^Research Center for Differentiation and Development of TCM Basic Theory, Jiangxi University of Traditional Chinese Medicine, Nanchang, China

## Abstract

*Objectives. *Acupuncture has been widely used to relieve chronic pain-related insomnia (CPRI). However, the efficacy of acupuncture for CPRI is uncertain. The purpose of this study was to evaluate the efficacy of acupuncture for CPRI.* Methods.* Seven electronic databases were searched from inception to December 2018. Randomized controlled trials (RCTs) were included if acupuncture was compared to sham acupuncture or conventional drug therapies for treating CPRI. Two reviewers screened each study and extracted data independently. Statistical analyses were conducted by RevMan 5.3 software.* Results.* A total of nine studies involving 944 patients were enrolled. The pooled analysis indicated that acupuncture treatment was significantly better than control group in improving effective rate (OR = 8.09, 95%CI = [4.75, 13.79],* P *< 0.00001) and cure rate (OR = 3.17, 95%CI = [2.35, 4.29],* P* < 0.00001), but subgroup analysis showed that there was no statistically significant difference between acupuncture and sham acupuncture in improving cure rate (OR =10.36, 95% CI [0.53, 201.45],* P*=0.12) based on one included study. In addition, meta-analysis demonstrated that acupuncture group was superior to control group in debasing PSQI score (MD = -2.65, 95%CI = [-4.00, -1.30],* P* = 0.0001) and VAS score (MD = -1.44, 95%CI = [-1.58, -1.29],* P* < 0.00001). And there was no significant difference in adverse events (OR =1.73, 95%CI = [0.92, 3.25],* P* =0.09) between the two groups.* Conclusions*. Acupuncture therapy is an effective and safe treatment for CPRI, and this treatment can be recommended for the management of patients with CPRI. Due to the low quality and small sample size of the included studies, more rigorously designed RCTs with high quality and large sample size are recommended in future.

## 1. Introduction

Chronic pain (CP), is defined as pain that is present for at least three months (International Association for the Study of Pain [IASP]). According to statistics, the incidence of CP in adults is about 10% to 25% [1], and the prevalence of CP is increasing as the population ages [2]. CP exerts tremendous pressure not only on individuals and families, but also on health systems and social economy. It is estimated that the annual cost of CP treatment and care in the United States is approximately $650 billion [3]. In addition, CP has been ranked the top cause of impacting the quality of life and shortening life-year in people, ahead of recognized depression, anxiety disorders, high blood pressure, and coronary heart disease [4, 5]. Reportedly, the majority of patients with CP report poor sleep quality [6, 7].

Insomnia, a common form of sleep disorder in CP patients, may directly lead to poor long-term results by affecting several aspects of the pathophysiology and psychosocial function of CP patients. In the United States, about 63% of CP patients have reported insomnia and were three times more likely to be diagnosed with insomnia [8, 9]. This striking level of comorbidity has suggested that the relationship between CP and sleep may be bidirectional [6, 7]. That is, pain may disturb the continuity of sleep, and insufficient sleep may reduce the tolerance of patients with CP [10]. Although the mechanism of CPRI is unclear, it is generally accompanied by an increase in excitability of the nervous system, so most current international guidelines recommend pharmacological management for CPRI relief, including sedative, anticonvulsants, opioids, and anti-inflammatory drugs (NSAIDs) [11]. Although most of these pharmacological treatments are available, their long-term clinical use is limited by significant side effects, such as addiction, allergic reactions, dizziness, and gastrointestinal reactions. In addition, cognitive-behavioral therapy (CBT) is considered a first-line psychotherapy for CPRI [12]. However, studies have shown that cognitive-behavioral therapy and other psychological interventions have limited regulatory effects [13].

Acupuncture, an alternative nonpharmacological treatment by stimulating specific acupuncture points on the body, has been widely used in clinical treatment [14], especially those involving neuroendocrine pathological changes, such as chronic pain, menopause, depression, and insomnia [15, 16]. Acupuncture can regulate neuroendocrine function by stimulating acupuncture points, such as increasing the release of endogenous opioid peptides to achieve analgesic effect [17] and increasing the expression of melatonin in pineal gland to improve sleep efficiency [18]. In many published clinical studies on acupuncture treatment of CPRI, most studies have shown that acupuncture is a reliable treatment for CPRI [9, 19].

There have been many systematic reviews of acupuncture treatment for insomnia [20]. However, there is still a lack of systematic review of CPRI, and the effectiveness of acupuncture in the treatment of CPRI is unclear. Therefore, we conducted this study to assess the efficacy and safety of acupuncture for CPRI.

## 2. Methods

### 2.1. Search Strategy

Two reviewers (Jianyu You, Ting Fang) independently performed a comprehensive literature search from multiple electronic databases, including EMBASE, PubMed, the Cochrane Library (Issue 12, 2018), the China National Knowledge Infrastructure (CNKI), the China Science and Technology Journal Database (VIP), the Chinese Biomedical Literature Database (CBM), and the WanFang databases from inception to December 20, 2018. The search strategy consists of three concept blocks: chronic pain (e.g., neck pain and back pain), Insomnia (e.g., insomnia and sleep disturbance), and acupuncture (e.g., acupuncture and needling) (see Supplementary Appendix 1 for the complete search terms). Then, we browsed the abstracts and full-text articles, respectively, and picked the eligible studies in line with the inclusion criteria. Additionally, all the available studies related to CPRI treatments were manually checked for any additional possibly relevant RCTs.

### 2.2. Inclusion and Exclusion Criteria

Relevant studies were included if the following criteria were met: (1) the included trials were RCTs studying acupuncture for treating CPRI; (2) the included patients were diagnosed with CPRI, regardless of the nationality, race, age, sex, or causes of pain (neck pain, back pain, shoulder pain, musculoskeletal pain, osteoarthritis, etc.); (3) studies on acupuncture compared to sham acupuncture or conventional pharmacological therapies were included. Here acupuncture only included manual acupuncture, excluding electroacupuncture, acupoint injection, acupoint application, auricular acupoint therapy, moxibustion, bloodletting cupping, etc.; (4) the primary outcome measures included Chinese medical efficacy standard (including effective rate and cure rate), the Pittsburgh Sleep Quality Index score (PSQI), and the visual analogue scale (VAS); the secondary outcome were adverse events in the acupuncture group and control group to assess safety; (5) full text should be available.

Studies with the following situations were excluded: (1) non-RCTs, (2) studies of acute pain or postsurgical pain and cancer pain, (3) wrong interventions, (4) duplicate studies, and (5) animal experiments.

### 2.3. Data Extraction

Two reviewers (Jianyu You, Ting Fang) independently extracted relevant data from the eligible studies, and then cross checked. If there are any uncertainties, we will solve them by discussing or consulting the other reviewer (Fushui Liu). The key information mainly included first author, year of publication, study location, baseline characteristics for participants, sample size, intervention, duration of intervention, randomization method, allocation concealment, blinding method, follow-up, dropout and withdrawal, outcome measurement indexes, and adverse events. If there is any ambiguous information in some of the studies, we attempted to contact the first author for more information by phone or email.

### 2.4. Quality Assessment

Two reviewers (Jianyu You, Ting Fang) independently assessed the quality and risk of bias (ROB) of the included literature by using the Cochrane Systematic Review Manual (version 5.1.0) RCT bias risk assessment tool. Disagreements would be resolved through discussion. The contents include the following: (1) random sequence generation, (2) allocation concealment, (3) blinding of participants and personnel, (4) blinding of outcome assessment, (5) incomplete outcome data, (6) selective reporting, and (7) other sources of bias.

### 2.5. Statistical Analysis

The meta-analysis was performed using Reviewer Manager Software, version 5.3. We defined *P* ≤ 0.05 as statistically significant between studies. For the categorical data (effective rate, cure rate and adverse events), we calculated combined odds ratio (OR) with 95% confidence intervals (CI); for continuous variables (PSQI and VAS), we estimated combined mean difference (MD) with 95% CI. The studies' heterogeneity was evaluated by Chi-square test and Higgins* I*^*2*^ test; when *I*^2^ ≤ 50% and *P* ≥ 0.10, the fixed effect model was used; otherwise, the random effect model was applied. Sensitivity analysis was used to assess the impact of the included trials on the final outcome. And Egger's test was performed using Stata14.0 statistical software to analyze potential publication bias. If *P* < 0.05, this was considered statistically significant.

## 3. Results

### 3.1. Literature Search Results

**Figure 1:**
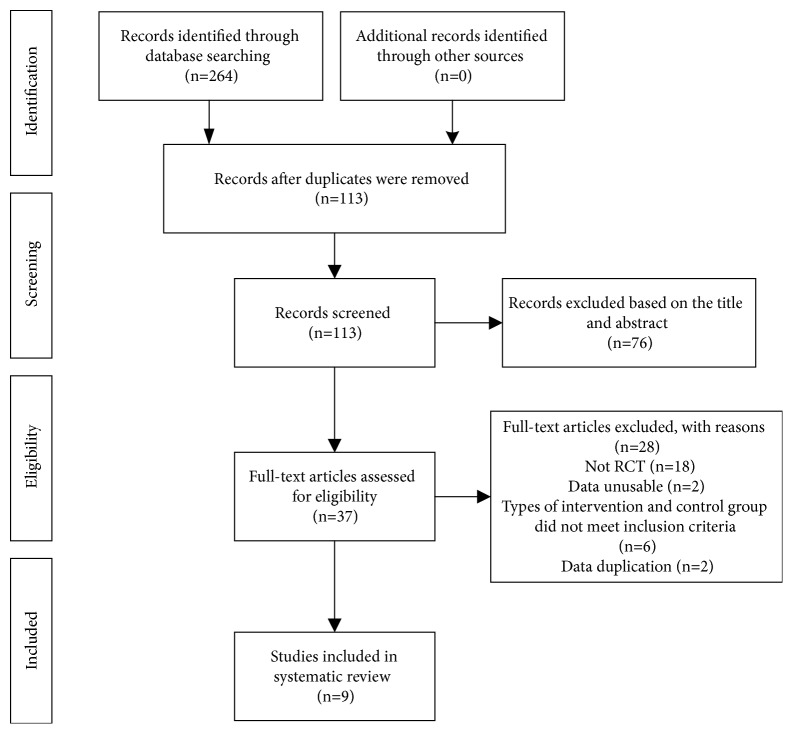
Flow diagram of the study.

A total of 264 studies were identified at the initial search. 113 studies remained after we excluded 151 duplicates with EndNote X7 software. Then, after scanning the title and abstracts, 76 studies were eliminated. Finally, 9 RCTs [19, 21–28] met our inclusion criteria after scanning full texts. The whole process of study selection is showed in Figure 1.

### 3.2. Basic Characteristics of Eligible Studies

The basic characteristics of all included studies were provided in Table 1. All studies were published between 2005 and 2018. The language of studies was English or Chinese. The sample size ranged from 12 to 272 participants. One article [19] originated in the United States and 8 [21–28] were from China. There were seven studies [21–23, 25–28] compared acupuncture with drugs, and the other two studies [19, 24] compared acupuncture with sham acupuncture.

### 3.3. Quality Assessment

**Figure 2:**
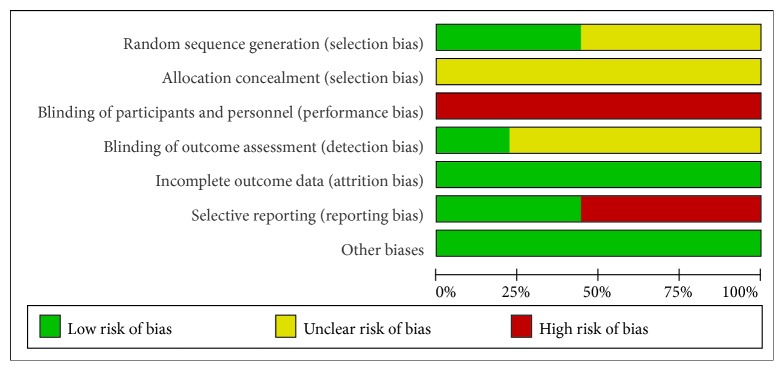
Risk of bias graph: review authors' judgements about each risk of bias item presented as percentages across all included studies.

**Figure 3:**
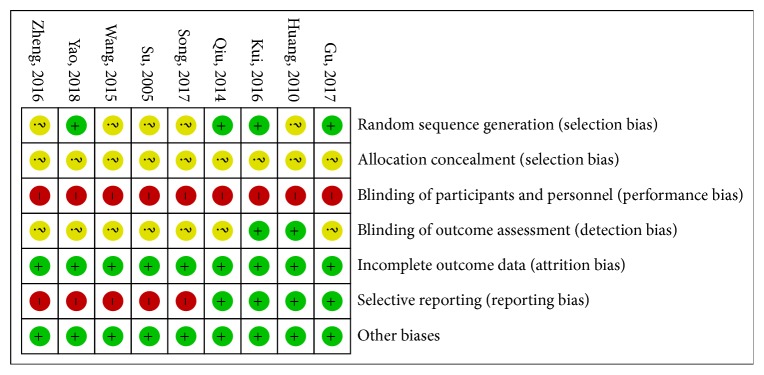
Risk of bias summary: review authors' judgments about each risk of bias item for each included study.

The risk of bias (ROB) results is shown in Figures 2 and 3. Three studies [22, 26, 28] used a random number table for generation random sequence and one study [24] was randomized according to the order of admission. The rest of studies only mentioned “random.” No study mentioned the use of allocation concealment. It is very difficult to blind therapists of acupuncture. Only two studies [19, 24] reported the blinding details about outcome assessment. There were two studies [19, 24] that reported dropout numbers and reasons, and the other studies reported no missing data. Five studies [21, 23, 25, 27, 28] did not report any details about adverse events.

### 3.4. Effective Rate

**Figure 4:**
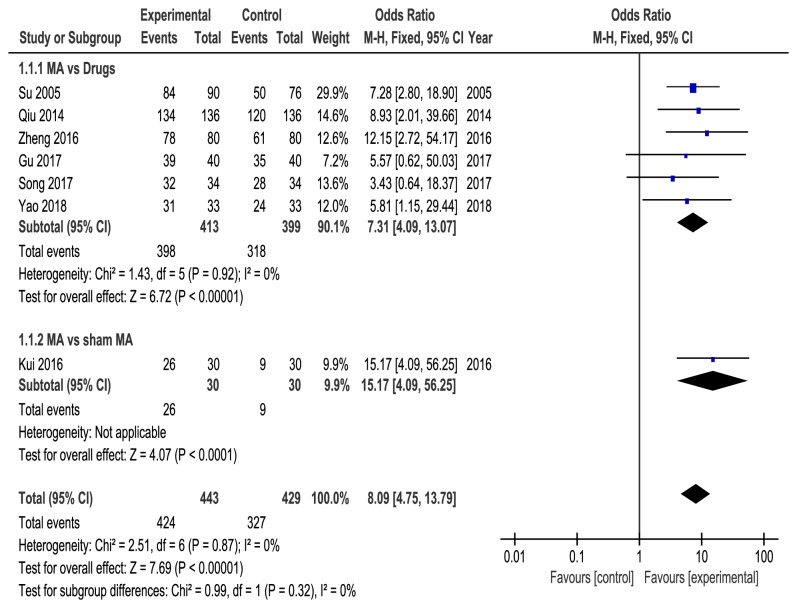
Meta-analysis on the total effective rate of acupuncture versus control group.

Seven RCTs [21, 22, 24–28] reported the effective rate of acupuncture. Six studies [21, 22, 25–28] compared acupuncture with drugs, and one study [24] compared acupuncture with sham acupuncture. Fixed effect model was used with no heterogeneity (*P* = 0.87, *I*^2^ = 0%), and our pooled results showed that acupuncture was more effective than control group (OR = 8.09, 95%CI = [4.75, 13.79],* P* < 0.00001) in improving effective rate. Subgroup analysis also showed that acupuncture is statistically significantly better than drugs (OR = 7.31, 95% CI = [4.09, 13.07], p < 0.00001, heterogeneity:* P* = 0.92, *I*^2^ = 0%) and sham acupuncture (OR = 15.17, 95% CI = [4.09, 56.25],* P* < 0.0001) (Figure 4).

### 3.5. Cure Rate

**Figure 5:**
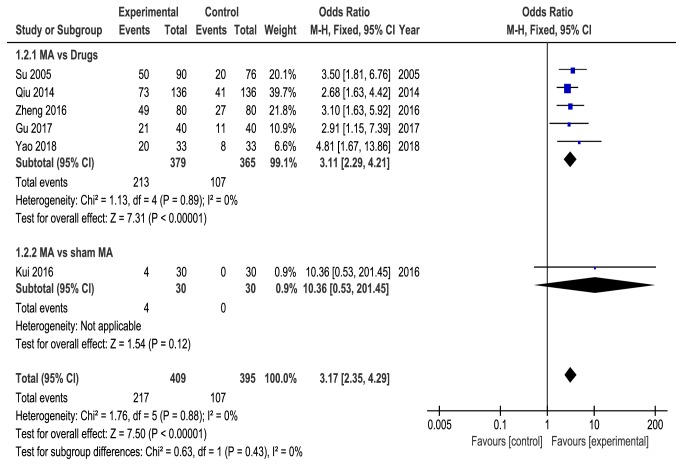
Meta-analysis on the cure rate of acupuncture versus control group.

Six studies [21, 22, 24–26, 28] reported the cure rate of acupuncture, five studies [21, 22, 25, 26, 28] compared acupuncture with drugs, and one study [24] compared acupuncture with sham acupuncture. No heterogeneity was found (*P* = 0.88, *I*^2^ = 0%), and the fixed-effects model showed that acupuncture was higher than control group (OR = 3.17, 95%CI = [2.35, 4.29],* P* < 0.00001) in improving cure rate. Subgroup analysis showed that acupuncture was statistically significantly better than drugs (OR = 3.11, 95% CI = [2.29, 4.21],* P* < 0.00001, heterogeneity:* P* = 0.89, *I*^2^ = 0%). However, there was no statistically significant difference between acupuncture and sham acupuncture based on one study (OR =10.36, 95% CI [0.53, 201.45],* P*=0.12) (Figure 5).

### 3.6. PSQI Score

**Figure 6:**
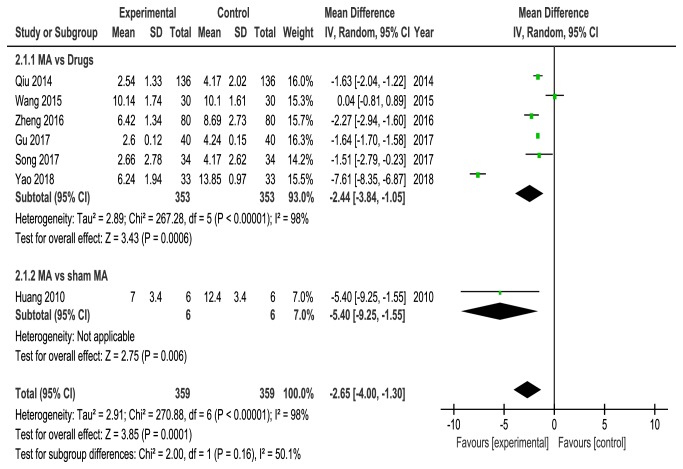
Meta-analysis for PSQI score of acupuncture versus control group.

Seven studies [19, 22, 23, 25–28] evaluated quality of sleep by using the PSQI score. Six studies [22, 23, 25–28] compared acupuncture with drugs, and one study [19] compared acupuncture with sham acupuncture. Data extracted showed obvious heterogeneity among these RCTs (*P* < 0.00001,* I*^*2*^ = 98%), a random-effects model was used, and our pooled results showed that acupuncture could further improve sleep quality associated with CPRI compared with control group (MD = -2.65, 95%CI = [-4.00, -1.30],* P* = 0.0001). Subgroup analysis also showed that acupuncture is statistically significantly better than drugs (MD =-2.44, 95% CI = [-3.84, -1.05],* P* = 0.0006, heterogeneity:* P* < 0.00001,* I*^*2*^ = 98%) and sham acupuncture (MD = -5.40, 95% CI = [-9.25, -1.55],* P* = 0.006) (Figure 6).

### 3.7. VAS Score

**Figure 7:**
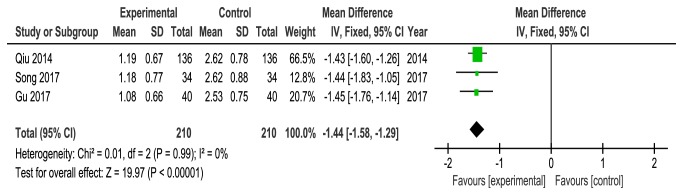
Meta-analysis for VAS score of acupuncture versus control group.

Three studies [22, 26, 27] compared acupuncture with drugs and reported the VAS score to measure pain intensity. Analysis of data from VAS score showed no heterogeneity (*P* = 0.99,* I*^*2*^ = 0%), and the fixed-effects model showed that acupuncture could further relieve pain associated with CPRI compared with drugs group (MD = -1.44, 95%CI = [-1.58, -1.29],* P* < 0.00001) (Figure 7).

### 3.8. Adverse Events

**Figure 8:**
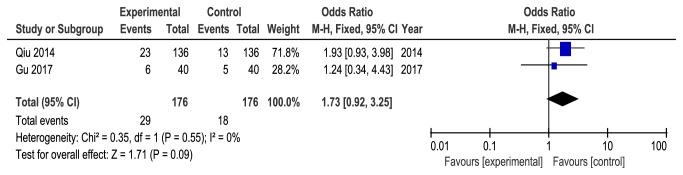
Meta-analysis for adverse events of acupuncture versus control group.

Four studies [19, 22, 24, 26] reported mild adverse events during the treatment, such as soreness or local bruising at needle site and nausea. Only two studies [22, 26] reported the exact number of adverse events. The others [19, 24] mentioned adverse events with no numbers. No heterogeneity was found between the two studies (*P* = 0.55,* I*^*2*^ = 0%), and the fixed-effects model showed no statistical difference in adverse events between the two groups (OR =1.73, 95%CI = [0.92, 3.25],* P* =0.09) (Figure 8).

### 3.9. Sensitivity Analysis and Publication Bias

Sensitivity analysis was used to evaluate the stability of Meta-analysis. For results from less than three studies, we performed sensitivity analysis by conversion effect models, such as adverse events; for the results of three or more studies, we performed sensitivity analysis by excluding the included studies one by one, such as effective rate, cure rate, PSQI score, and VAS score. Sensitivity analysis showed that the results of each Meta-analysis were stable. Furthermore, owing to an insufficient number of included studies (fewer than 10 studies), we analyzed publication bias through Egger's test, and the results showed that there was no publication bias in total effective rate, PSQI scores, and VAS scores (*P* > 0.05). But there was a publication bias in the cure rate (*P* < 0.05), which may be caused by the fact that the negative results were not published or the grey literature was not included in the study (Tables 2 and 3).

## 4. Discussion

Insomnia is one of the most major public health problems. In general, insomnia is divided into primary insomnia and secondary insomnia. Primary insomnia is defined as insomnia without comorbidity. Secondary insomnia is also called “comorbid insomnia” and is a term used when insomnia is associated with another disease, such as pain, depression, anxiety, coronary heart disease, and other conditions [29, 30]. In this study, CPRI is a category of comorbid insomnia associated with CP, with clinical manifestations of CP and insomnia. In fact, CP and insomnia are common diseases with high incidence and often co-exist. Although the reciprocal relationship between CP and insomnia is unclear, both adversely affect the patient's life and work [31]. In addition, recent relevant systematic reviews have shown that there is an interaction between pain and insomnia, which means that pain increases the risk of insomnia in patients and vice versa [32]. Due to the unremitting nature of CP, it is difficult for a painful patient to fall asleep quickly or to maintain a long sleep compared to a healthy person. Simultaneously, insufficient sleep not only weakens people's immune function, affecting people's ability to regulate emotions, but also increases pain sensitivity [33]. In this way, a vicious circle is formed between CP and insomnia.

Acupuncture is part of Traditional Chinese Medicine (TCM). It achieves the effect of treating diseases by inserting acupuncture needles into specific acupuncture points [34]. Acupuncture has a good analgesic effect and is easy to operate, safe, and economical [35]. Acupuncture as a treatment for pain relief is widely recognized in many countries around the world, such as the United States, Australia, Canada, and the United Kingdom. In the United States, acupuncture is one of the few traditional medical treatments with a national licensure system [34]. Additionally, Research on acupuncture analgesia has a long history. Acupuncture analgesia is a complex network, from the periphery to the center, involving the entire nervous system. Experimental studies have shown that the mechanism of acupuncture analgesia is the result of a combination of various biologically active molecules in the pain process, including neurotransmitters, neuromodulators, neuropeptides, cell signaling molecules and inflammatory mediators [35, 36]. Simultaneously, relevant sleep research also fully affirmed the sedative and hypnotic effects of acupuncture and made preliminary progress. Recent studies provide laboratory based evidence that acupuncture affects the sleep-wake-up cycle by interfering with many levels of insomnia-related cytokines and neurotransmitters (such as 5-HT, NE, DA, GABA, Glu, IL-1, IL-6, TNF, and NO) which plays a role in sedative hypnosis and promotes sleep [37–40].

In our current study, we pooled the data from nine studies involving 944 patients. Our pooled analysis indicated that acupuncture treatment was significantly better than drugs group in improving effective rate and cure rate and in debasing PSQI score and VAS score. In addition, compared with sham acupuncture, manual acupuncture treatment has an advantage in improving the effective rate and reducing the PSQI score, but the two groups have no statistical significance in improving the cure rate. In this meta-analysis, only four studies reported relevant adverse events. The combined data showed no significant difference in adverse reactions between acupuncture group and control group. Therefore, we can carefully recommend that acupuncture is as safe as control group for CPRI. In addition, adverse events were relatively mild, mainly including bruises, soreness, nausea, dizziness, and other discomforts. These adverse events can be effectively avoided by strengthening the aseptic operation specification, and improving the professional ability of doctors. Based on the findings of our included studies, we propose that acupuncture is an effective and safe therapy for patients with CPRI.

## 5. Conclusions

The results of our current systematic review and meta-analysis suggested that acupuncture therapy is an effective and safe treatment for patients with CPRI, and it can be recommended for the management of CPRI. However, due to insufficient number of included studies, low methodological quality and heterogeneity of results, more large-scale and high-quality RCTs are needed to further investigate the therapeutic effect of acupuncture for CPRI.

## Figures and Tables

**Table 1:** Characteristics of included studies.

Study	Study location	Sample size (male/female)	Interventions	Treatment period	Outcomes	Adverse events
Su (2005)	Guangdong, China	AG: 26/64 CG: 20/56	AG: MA CG: drugs (Estazolam)	AG: once a day for 12 days, 30 min CG: twice a day for 12 days	CE	NR
Huang (2010)	USA	AG: 6 CG: 6	AG: MA CG: sham acupuncture	Two times per week for 4 weeks, followed by one time per week for 4 weeks, 30 min	PSQI	Not serious, mild
Qiu (2014)	Beijing, China	AG: 71/65 CG: 74/62	AG: MA CG: drugs (Eszopiclone)	AG: once a day for 30 days, 30 min CG: once a day for 30 days	CE PSQI VAS	Not serious, mild
Wang (2015)	Sichuan, China	AG: 30 CG: 30	AG: MA CG: drugs (Estazolam)	AG: once a day for 20 days, 30 min CG: once a day for 20 days	PSQI	NR
Kui (2016)	Guangdong, China	AG:16 /14 CG:15 /15	AG: MA CG: sham acupuncture	Once a day for 2 weeks, 30 min	CE	Not serious, mild
Zheng (2016)	Guangdong, China	AG: 44/36 CG: 43/37	AG: MA CG: drugs (Zaoren Anshen capsule)	AG: once a day for 20 days, 30 min CG: once a day for 20 days	CE PSQI	NR
Gu (2017)	Zhejiang, China	AG: 24/16 CG: 22/18	AG: MA CG: drugs (Alprazolam)	AG: once a day for 4 weeks, 30 min CG: once a day for 4 weeks	CE PSQI VAS	Not serious, mild
Song (2017)	Neimenggu, China	AG: 18/16 CG: 19/15	AG: MA CG: drugs (Estazolam)	AG: once a day for 30 days, 30 min CG: once a day for 30 days	CE PSQI VAS	NR
Yao (2018)	Yunnan, China	AG: 10/23 CG: 10/23	AG: MA CG: drugs (Eszopiclone)	AG: once a day for 20 days, 30 min CG: once a day for 20 days	CE PSQI	NR

AG, acupuncture group; CG, control group; CE, clinical effect; MA, manual acupuncture; NR, not reported; PSQI, the Pittsburgh Sleep Quality Index score; VAS, the visual analogue scale.

**Table 2:** Summary of sensitivity analysis for the clinical effect.

	OR fluctuation	95% CI fluctuation	Publication bias (P value)
Effective rate	(7.31, 8.83)	(4.09, 16.05)	0.569
Cure rate	(3.06, 3.49)	(2.20, 5.11)	0.04

Note: *P* < 0.05 indicates that a publication bias exists.

**Table 3:** Summary of sensitivity analysis of parameters for PSQI score and VAS score.

	MD fluctuation	95% CI fluctuation	Publication bias (P value)
PSQI score	(-3.14, -1.54)	(-5.20, -0.68)	0.406
VAS score	(-1.45, -1.43)	(-1.69, -1.20)	0.407

Note: *P* < 0.05 indicates that a publication bias exists.

## Additional Points

*Study Limitations.* However, several limitations had existed in our study as follows: first, insufficient number of included studies and small sample sizes may lead to inaccurate evidence in our research. Second, most of the included studies generally have low methodological quality, only four studies described the random method, no study mentioned the use of allocation concealment, and only two studies reported the blinding details that might limit the accuracy of the conclusions of this study. Third, the primary outcome of clinical effect standard (including effective rate and cure rate) for CPRI used in meta-analysis is not an internationally accepted standard; it was classified into clinical cure, markedly effective, effective, and ineffective according to the patient's subjective feeling rather than quantitative criteria; it remained debatable. Last, only a few studies have reported details of adverse events; thus, the safety of acupuncture for CPRI remains to be further explored. In view of the above limitations, it is recommended to use more rigorous large-scale and well-designed RCTs to provide higher quality evidence and to evaluate the efficacy of acupuncture in the treatment of CPRI.

## Supplementary Materials

Supplementary Materials Supplemental materials explain the complete search strategy. Our search strategy consists of three concept blocks: chronic pain (e.g., neck pain and back pain), Insomnia (e.g., insomnia and sleep disturbance), and acupuncture (e.g., acupuncture and needling); we conducted a Mesh term search for each concept block.

## References

[1] Mathias J. L., Cant M. L., Burke A. L. J. (2018). Sleep disturbances and sleep disorders in adults living with chronic pain: a meta-analysis. *Sleep Medicine*.

[2] Ramage-Morin P. L. (2008). Chronic pain in Canadian seniors. *Health Reports*.

[3] Gaskin D. J., Richard P. (2012). The economic costs of pain in the United States. *The Journal of Pain*.

[4] Fernández A., Saameño J. Á., Pinto-Meza A. (2010). Burden of chronic physical conditions and mental disorders in primary care. *The British Journal of Psychiatry*.

[5] Tang N. K., Lereya S. T., Boulton H., Miller M. A., Wolke D., Cappuccio F. P. (2015). Nonpharmacological treatments of insomnia for long-term painful conditions: a systematic review and meta-analysis of patient-reported outcomes in randomized controlled trials. *Sleep*.

[6] Alsaadi S. M., McAuley J. H., Hush J. M. (2014). The bidirectional relationship between pain intensity and sleep disturbance/quality in patients with low back pain. *The Clinical Journal of Pain*.

[7] Finan P. H., Goodin B. R., Smith M. T. (2013). The association of sleep and pain: an update and a path forward. *The Journal of Pain*.

[8] Knutson K. (2015). Sleep and pain: summary of the 2015 sleep in america poll. *Sleep Health*.

[9] Garner B. K., Hopkinson S. G., Ketz A. K., Landis C. A., Trego L. L. (2018). Auricular acupuncture for chronic pain and insomnia: a randomized clinical trial. *Medical Acupuncture*.

[10] Tang H.-Y., Vitiello M. V., Perlis M., Mao J. J., Riegel B. (2014). A pilot study of audio–visual stimulation as a self-care treatment for insomnia in adults with insomnia and chronic pain. *Applied Psychophysiology and Biofeedback*.

[11] Elvir-Lazo O. L., White P. F. (2010). The role of multimodal analgesia in pain management after ambulatory surgery. *Current Opinion in Anaesthesiology*.

[12] Pigeon W. R., Moynihan J., Matteson-Rusby S. (2012). Comparative effectiveness of CBT interventions for co-morbid chronic pain insomnia: a pilot study. *Behaviour Research and Therapy*.

[13] Ehde D. M., Dillworth T. M., Turner J. A. (2014). Cognitive-behavioral therapy for individuals with chronic pain: efficacy, innovations, and directions for research. *American Psychologist (Salma)*.

[14] Bauer B. A., Tilburt J. C., Sood A., Li G.-X., Wang S.-H. (2016). Complementary and alternative medicine therapies for chronic pain. *Chinese Journal of Integrative Medicine*.

[15] Chen S., Wang S., Rong P. (2014). Acupuncture for visceral pain: neural substrates and potential mechanisms. *Evidence-Based Complementary and Alternative Medicine*.

[16] Huo Z., Guo J., Li D. (2013). Effects of acupuncture with meridian acupoints and three Anmian acupoints on insomnia and related depression and anxiety state. *Chinese Journal of Integrative Medicine*.

[17] Harris R. E., Zubieta J.-K., Scott D. J., Napadow V., Gracely R. H., Clauw D. J. (2009). Traditional Chinese acupuncture and placebo (sham) acupuncture are differentiated by their effects on *μ*-opioid receptors (MORs). *Neuroimage*.

[18] Spence D. W., Kayumov L., Chen A. (2004). Acupuncture increases nocturnal melatonin secretion and reduces insomnia and anxiety: a preliminary report. *The Journal of Neuropsychiatry and Clinical Neurosciences*.

[19] Huang W., Bliwise D. L., Carnevale C. V., Kutner N. G. (2010). Acupuncture for pain and sleep in knee osteoarthritis. *Journal of the American Geriatrics Society*.

[20] Huang W., Kutner N., Bliwise D. L. (2009). A systematic review of the effects of acupuncture in treating insomnia. *Sleep Medicine Reviews*.

[21] Su X., Wu Z. Q. (2005). Clinical observation on 90 cases of acupuncture in the treatment of cervical insomnia. *Journal of New Chinese Medicine*.

[22] Qiu X. L. (2014). Acupuncture treating 136 cases of nerve root type of cervical disease with severe sleep disorders. *World Chinese Medicine Journal*.

[23] Wang X. X., Ma J., Chen H. L. (2015). Clinical study on acupuncture treatment of cervical insomnia. *Journal of Traditional Chinese Medicine*.

[24] Kui Y., Chen X.-H., Xu Z.-H. (2016). Clinical study on chen's flying acupuncture in the treatment of insomnia combined with vertebral artery type of cervical spondylosis. *Journal of Chinese General Practice*.

[25] Zheng L. H., Yu X. M., Liu Y. X. (2016). Clinical observation on 80 cases of acupuncture in the treatment of cervical insomnia. *Chinese Journal of Ethnomedicine and Ethnopharmacy*.

[26] Gu J. J. (2017). Comparative study of acupuncture treatment and conventional drug treatment on cervical disease with severe sleep disorders. *Chinese Journal of Primary Medicine and Pharmacy*.

[27] Song Y. D., Tian W. L. (2017). Clinical efficacy and safety of acupuncture in the treatment of cervical spondylotic radiculopathy with severe sleep disturbance. *Journal of Clinical Medical Literature*.

[28] Yao B. (2018). *Clinical research on the neck cavity as the main acupuncture treatment for the cervical source insomnia*.

[29] National Institutes of Health (2005). National institutes of health state of the science conference statement on manifestations and management of chronic insomnia in adults. *Sleep*.

[30] Zhao H., Li D., Yang Y., Liu Y., Li J., Mao J. (2019). Auricular plaster therapy for comorbid insomnia: a systematic review and meta-analysis of randomized controlled trials. *Evidence-Based Complementary and Alternative Medicine*.

[31] Dragioti E., Bernfort L., Larsson B., Gerdle B., Levin L. Å. (2018). Association of insomnia severity with well-being, quality of life and health care costs: A cross-sectional study in older adults with chronic pain (PainS65+). *European Journal of Pain*.

[32] Kelly G. A., Blake C., Power C. K. (2011). The association between chronic low back pain and sleep: a systematic review. *Clinical Journal of Pain*.

[33] Finan P. H., Buenaver L. F., Runko V. T., Smith M. T. (2014). Cognitive-behavioral therapy for comorbid insomnia and chronic pain. *Sleep Medicine Clinics*.

[34] Lim T., Ma Y., Berger F., Litscher G. (2018). Acupuncture and neural mechanism in the management of low back pain—an update. *Medicines (Basel)*.

[35] Li Y., Wu F., Cheng K. (2018). Mechanisms of acupuncture for inflammatory pain. *Zhen Ci Yan Jiu*.

[36] Zhang R., Lao L., Ren K., Berman B. M. (2014). Mechanisms of acupuncture-electroacupuncture on persistent pain. *Anesthesiology*.

[37] Sahu S., Ray K., Yogendra Kumar M. (2012). Valeriana wallichii root extract improves sleep quality and modulates brain monoamine level in rats. *Phytomedicine*.

[38] He B., Bi K., Jia Y. (2013). Rapid analysis of neurotransmitters in rat brain using ultra-fast liquid chromatography and tandem mass spectrometry: application to a comparative study in normal and insomnic rats. *Journal of Mass Spectrometry*.

[39] Churchill L., Taishi P., Wang M. (2006). Brain distribution of cytokine mRNA induced by systemic administration of interleukin-1*β* or tumor necrosis factor *α*. *Brain Research*.

[40] Chen P. D., Yang Z. X., LI J. Y. (2015). Mechanism research advances of acupuncture in the treatment of insomnia. *China Medical Herald*.

