# Supplementary material for: Acupuncture for Chronic Pain-Related Insomnia: A Systematic Review and Meta-Analysis
Source: Evid Based Complement Alternat Med. 2019 Jun 24;2019:5381028. doi: 10.1155/2019/5381028 (PMC6612974; doi:10.1155/2019/5381028)
Supplement: Supplementary Materials — Supplemental materials explain the complete search strategy. Our search strategy consists of three concept blocks: chronic pain (e.g., neck pain and back pain), Insomnia (e.g., insomnia and sleep disturbance), and acupuncture (e.g., acupuncture and needling); we conducted a Mesh term search for each concept block. [file 5381028.f1.pdf]

Supplementary Material

Literature search strategy

The search algorithm was constructed as follows:

#1 Mesh term: (acupuncture OR needling OR acupoint OR acupressure)

#2 Mesh term: (insomnia OR sleep OR sleep disturbance OR sleep disorder OR sleepless)

#3 Mesh term: (pain OR chronic pain OR long-lasting pain OR intermittent pain OR long-term pain OR persistent pain OR chronic noncancer pain OR neuropathic pain OR neuralgia OR intractable pain OR nociceptive pain OR whiplash injuries OR rheumatoid OR musculoskeletal pain OR myalgia OR fibromyalgia OR myofascial pain OR migraine OR headache OR muscle OR tendon OR ligament OR skeletal OR bone OR cartilage OR spine OR cervical vertebrae OR thoracic vertebrae OR lumbar vertebrae OR sacrum OR coccyx OR intervertebral disc OR ankylosing spondylitis OR arthritis OR arthralgia OR polyarthritis OR joint OR osteoarthritis OR rheumatoid arthritis OR neck pain OR shoulder pain OR back pain OR backache OR low back pain OR pelvic girdle pain OR upper extremity pain OR arm pain OR elbow pain OR forearm pain OR hand pain OR lower extremity pain OR hip pain OR knee pain OR foot pain OR heel pain OR metatarsalgia OR leg pain OR lumbosacral region pain OR sacrococcygeal region pain)

#4: 1 AND 2 AND 3

Note: When searching the Chinese databases, we used Chinese key words and translated them in this article.
